# Potential therapeutic GSK-3β inhibitor 9-ING-41 is active in combination with venetoclax in double-hit lymphoma (DHL)

**DOI:** 10.1080/15384047.2025.2581831

**Published:** 2025-11-11

**Authors:** Haohao Lei, Yunxia Zhang, Haiqing Zheng, Pengcheng Shi, Xiaolei Wei, Xutao Guo

**Affiliations:** a Department of Hematology, Nanfang Hospital, Southern Medical University, Guangzhou, Guangdong, P.R. China; b Department of Hematology, Huadu Institute of Medical Sciences, Huadu District People’s Hospital of Guangzhou, Guangzhou, Guangdong, P.R. China; c Department of Nosocomial Infection Management, Nanfang Hospital, Southern Medical University, Guangzhou, Guangdong, P.R. China; d Clinical Medical Research Center of Hematological Diseases of Guangdong Province, Guangzhou, P.R. China

**Keywords:** Glycogen synthase kinase-3, double-hit lymphoma, 9-ING-41, apoptosis, cell cycle arrest

## Abstract

**Background:**

Double-hit lymphoma (DHL) exhibits aggressive behavior due to dysregulated proliferation and resistance to apoptosis. Current therapies, including R-CHOP, show limited efficacy, necessitating novel strategies. 9-ING-41, a novel ATP-competitive small-molecule inhibitor that targets glycogen synthase kinase-3β (GSK-3β), has emerged as a promising therapeutic agent because of its ability to disrupt oncogenic signaling pathways associated with tumor progression and treatment resistance. However, the antitumor effects of 9-ING-41 in DHL remain unclear.

**Materials and methods:**

DHL cell lines (Karpas-422 and SuDHL2) were treated with venetoclax and 9-ING-41, either alone or in combination. Cell viability in cytotoxicity assays was assessed using the CCK-8 assay, while apoptosis and cell cycle changes were analyzed via flow cytometry. Western blotting was employed to evaluate alterations in the levels of GSK-3β and WNT/β-catenin pathway proteins following treatment.

**Results:**

In preclinical studies utilizing DHL cell models, the single agent 9-ING-41 demonstrated robust biological activity through inducing significant G1/S phase cell cycle arrest and triggering apoptosis. When coadministered with venetoclax, a clinically approved BCL-2 inhibitor, the combination exhibited marked synergistic cytotoxicity in DHL cells, achieving superior inhibitory effects compared to either agent alone. The combined treatment enhanced cell cycle arrest, significantly reducing the number of S-phase cells and reinforcing G0/G1 arrest. Further mechanistic studies revealed that the combination modulated key proteins in the GSK-3 pathway and downstream WNT/β-catenin pathway, revealing a potential synergistic mechanism.

**Conclusion:**

The demonstrated single-agent efficacy and combination synergy with venetoclax support the potential of 9-ING-41 as a novel therapeutic strategy for DHL. These findings provide a proof-of-concept that may serve as a basis for future preclinical investigations in DHL.

## 1 Introduction

Double-hit lymphoma (DHL) is a distinct type of high-grade B-cell lymphoma (HGBL or HGBL-DH/TH) characterized by rearrangements in both the MYC and BCL2 genes.^1^ These genetic features lead to excessive cell proliferation and resistance to apoptosis, making DHL challenging to treat. The pathogenesis of DHL primarily involves abnormalities in the BCL2 and MYC genes.^2^^,^^3^ Overexpression of BCL2, caused by either BCL2 amplification or translocation, inhibits the apoptotic process, while MYC rearrangement promotes continuous cell proliferation.^4^ The combination of these effects results in uncontrolled cell proliferation and survival, resulting in highly malignant and difficult-to-treat tumors. Current treatments for DHL face significant challenges, mainly due to its low response to standard chemotherapy protocols and high relapse rate. Conventional treatments, such as standard-of-care chemoimmunotherapy with rituximab, cyclophosphamide, doxorubicin, vincristine, and prednisone (R-CHOP), result in limited cure rates for most DHL patients.^5^ Therefore, more effective drugs are needed to improve treatment outcomes for patients with DHL.

GSK-3, comprising the GSK-3α and GSK-3β subtypes, are serine/threonine kinases critical for various intracellular signaling pathways that influence cell growth, differentiation, and apoptosis. Their broad impact links them to numerous diseases. Researchers have developed numerous GSK-3-specific inhibitors for studies in cancer, cardiovascular diseases, diabetes, inflammation, neurodegenerative disorders, and mental health.^6^ GSK-3β is considered a potential tumor suppressor because it phosphorylates and targets pro-oncogenic molecules, including c-Jun, c-Myc, cyclin D1 and *β*-catenin, for ubiquitin-dependent proteasomal degradation.^7-9^ Over the past decade, GSK-3β has emerged as a therapeutic target in several different types of cancer,^10^ including lymphoma.^11^ 9-ING-41 is a GSK-3β inhibitor with notable antitumor activity. By targeting GSK-3, it inhibits tumor growth and induces apoptosis across various cancer models.^12^ Preliminary studies in hematologic malignancies indicate that 9-ING-41 holds potential as a candidate for future clinical evaluation, particularly in patients who have developed resistance to conventional therapies. The present study aimed to determine whether 9-ING-41 may potentiate the antitumor effects of chemotherapeutic drugs and targeted therapeutics and increase the cytotoxic effects on DHL cell lines.

## 2 Results

### 2.1 Treatment with 9-ING-41 inhibits the proliferation of DHL cells, and combined application of venetoclax and 9-ING-41 in DHL cells has significant synergistic effects on the inhibition of proliferation

**Figure 1.**
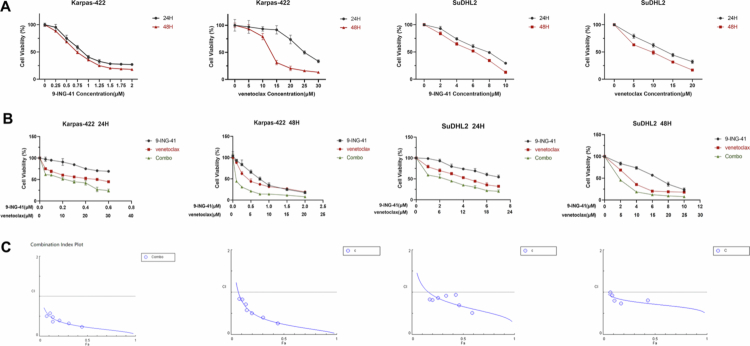
Treatment with 9-ING-41 inhibits the proliferation and survival of DHL cells. (A) Relative cell proliferation was measured by CCK-8 in DHL cells treated with the indicated doses of 9-ING-41 for 24 or 48 H. Differences were analyzed by one-way ANOVA. (B)(C) Concentration curve of the inhibitory effect of 9-ING-41 combined with venetoclax on the proliferation of DHL cells after 24 H and 48 H (B), and the combined index CI and the combined proliferation inhibition rate Fa were calculated and plotted via CompuSyn software (C). Combo represented 9-ING-41 and venetoclax.

After administering a single treatment of 9-ING-41 to DHL cell lines (Karpas-422 and SuDHL2) across an appropriate concentration gradient for durations of 24 hours and 48 hours, the OD values for each cell line at various concentrations and time points were assessed using the CCK-8 method. The cell survival rates were subsequently calculated. The experiment was independently replicated more than three times to ensure consistency. The results demonstrated that within a specific concentration range, the inhibitory effect of 9-ING-41 on the proliferation of DHL cell lines (Karpas-422 and SuDHL2) intensified as the concentration increased. The IC50 values for 9-ING-41 were determined following treatment periods of 24 hours and 48 hours. For Karpas-422 cells, the 24-hour IC50 value was 0.91 ± 0.04 μM, while the 48-hour IC50 value was 0.74 ± 0.02 μM. In contrast, for SUDHL2 cells, the 24-hour IC50 value was 7.16 ± 0.35 μM, and the 48-hour IC50 value was 5.44 ± 0.47 μM (Figure 1A).

The cell activity ratios (%) of Karpas-422 cells treated with 0.3 μM 9-ING-41 and 15 μM venetoclax for 24 hours were 84.60 ± 2.01% and 56.09 ± 2.56%, respectively. When these two drugs were combined, the cell activity ratio decreased significantly to 44.84 ± 3.38%. One-way ANOVA revealed a statistically significant difference between the combination therapy group and both the 9-ING-41 monotherapy group and the venetoclax monotherapy group (*P* < 0.0001). Similarly, when SuDHL2 cells were treated with 4 μM 9-ING-41 or 12 μM venetoclax, the cell activity ratios were 73.79 ± 2.57% and 53.05 ± 2.37%, respectively. When these drugs were combined, the cell activity ratio drastically decreased to 35.15 ± 1.33%. Two-way analysis of variance confirmed that this difference between the combination therapy group and the monotherapy groups was statistically significant (*P* < 0.0001). Notably, the effect observed in the 48-hour group was consistent with that in the 24-hour group (Figure 1B).

The CI (combination index) of 9-ING-41 in combination with venetoclax was computed using CompuSyn software. A CI value less than 1 indicates a synergistic effect between the two drugs, while a CI value equal to 1 suggests an additive effect. Conversely, a CI value greater than 1 implies antagonism between the drugs. For the DHL cell lines (Karpas-422 and SuDHL2), the CI value of the combination of 9-ING-41 and venetoclax was less than 1 at both 24 and 48 hours (Figure 1C).

### 2.2 Treatment with 9-ING-41 monotherapy and combination therapy has notable effects on cell cycle arrest in DHL cells

To evaluate the impact of 9-ING-41, venetoclax, and their combined use on DHL cell cycle progression, Karpas-422 and SuDHL2 cells were treated. After 48 hours, flow cytometry was used to analyze cell cycle arrest.

In Karpas-422 cells, 9-ING-41 monotherapy caused significant G2/M phase arrest (8.27 ± 1.77% vs. control's 22.97 ± 3.72%), while venetoclax monotherapy led to G0/G1 phase arrest (69.60 ± 0.90% vs. control's 43.00 ± 2.04%). Combination therapy further reduced the percentage of S-phase cells to 10.53 ± 0.32% and primarily blocked cells in the G0/G1 phase (74.27 ± 2.66%).

Similarly, in SuDHL2 cells, 9-ING-41 monotherapy caused G2/M phase arrest (11.71 ± 1.56% vs. control's 24.9 ± 4.12%), and venetoclax monotherapy led to G0/G1 phase arrest (66.9 ± 0.62% vs. control's 38.93 ± 1.89%). Compared with 9-ING-41 monotherapy, combination therapy reduced S-phase cells to 10.45 ± 0.78% and significantly increased G0/G1 phase arrest.

**Figure 2.**
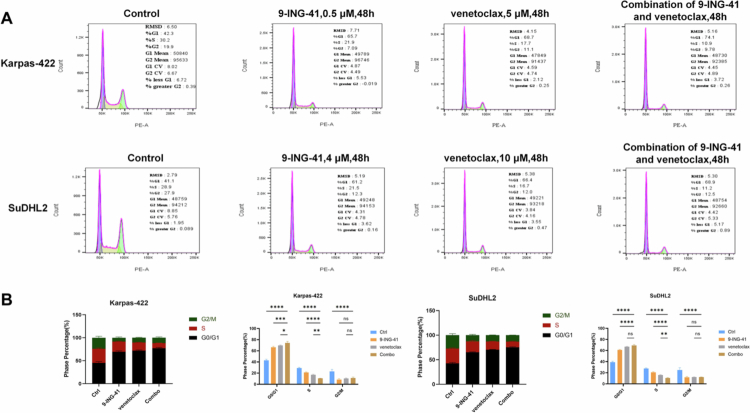
9-ING-41 induces cell cycle arrest in DHL cells. (A) Flow cytometry was performed in Karpas-422 cells treated with 0.5 μM 9-ING-41, 5 μM venetoclax, 0.5 μM 9-ING-41 combined with 5 μM venetoclax for 48 h. Flow cytometry was performed on the SuDHL2 cell line treated with 4 μM 9-ING-41, 10 μM venetoclax, or 4 μM 9-ING-41 combined with 10 μM venetoclax for 48 h. (B) Cell cycle distribution and rates of the sub-G1, G1, S and G2 populations are presented following the treatment of DHL cells with 9-ING-41 as indicated. *P < 0.05, **P < 0.01, ***P < 0.001, ****P < 0.0001, ns: no significance.

These results indicate that both monotherapy and combination therapy have notable cell cycle arrest effects, with combination therapy primarily blocking cells in the G0/G1 phase (Figure 2).

### 2.3 Effects of 9-ING-41, venetoclax, and their combination on the apoptosis in DHL cells

To assess the impact of 9-ING-41, venetoclax, and their combined administration on the apoptosis of DHL cells, we treated the Karpas-422 and SuDHL2 cell lines with various concentrations of these drugs and a control group of 0.1% DMSO. Forty-eight hours later, apoptosis was detected using flow cytometry with Annexin V-FITC/PI double staining.

**Figure 3.**
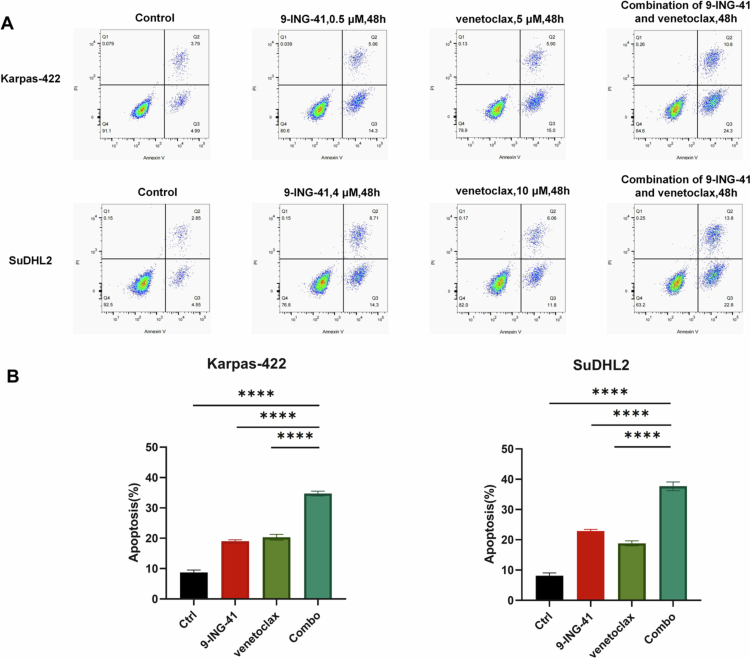
Effects of 9-ING-41, venetoclax, and their combination on apoptosis in DHL cells. (A) Flow cytometry was performed in Karpas-422 cells treated with 0.5 μM 9-ING-41, 5 μM venetoclax, 0.5 μM 9-ING-41 combined with 5 μM venetoclax for 48 h. Flow cytometry was performed in the SuDHL2 cell line treated with 4 μM 9-ING-41, 10 μM venetoclax, or 4 μM 9-ING-41 combined with 10 μM venetoclax for 48 h. (B) Apoptosis rates of DHL cells following the treatment with 9-ING-41, venetoclax, or their combination are shown as indicated. *P < 0.05, **P < 0.01, ***P < 0.001, ****P < 0.0001, ns: no significance.

The results indicated that for both the Karpas-422 and the SuDHL2 cell lines, the percentage of apoptotic cells significantly increased in the 9-ING-41 monotherapy group, the venetoclax monotherapy group, and the dual drug combination group compared to the control group. Further inter-group comparisons using the LSD method revealed that the apoptosis ratio in the dual-drug combination group was statistically significantly higher than that in the monotherapy groups (*P* < 0.0001), as illustrated in Figure 3.

### 2.4 Effect of venetoclax combined with 9-ING-41 on the expression of the GSK-3β protein family in the DHL cell lines

To investigate the effects of 9-ING-41, venetoclax, and their combined administration on the expression profiles of GSK-3β and proteins related to the WNT/β-catenin pathway in the DHL cell lines, we conducted the following experiments. The Karpas-422 cell line was treated with 0.1% DMSO as a control, 5 μM venetoclax, 0.5 μM 9-ING-41, or a combination of 5 μM venetoclax with 0.5 μM 9-ING-41. Similarly, the SuDHL2 cell line was treated with 0.1% DMSO as a control, 10 μM venetoclax, 4 μM 9-ING-41, or a combination of 10 μM venetoclax with 4 μM 9-ING-41. Forty-eight hours post-treatment, total protein was extracted from both cell lines and subjected to Western blot analysis to assess the expression levels of the target proteins.

**Figure 4.**
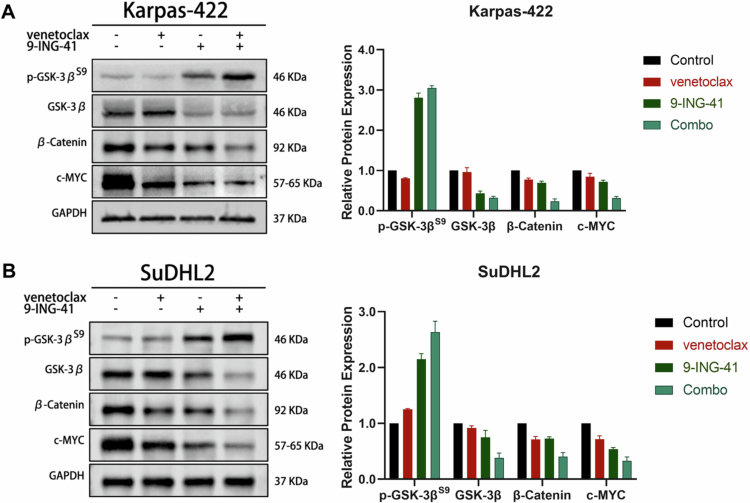
Effect of venetoclax combined with 9-ING-41 on the expression of the GSK-3β protein family in the DHL cell line. (A, B) Western blot analysis of the effects of venetoclax, 9-ING-41 alone, and in combination, on the expression of the GSK-3β protein family and WNT/β-catenin pathway-associated proteins in the Karpas-422 and SuDHL2 cell lines.

The results demonstrated that, in comparison with the control group, the 9-ING-41 monotherapy group and the venetoclax monotherapy group exhibited a decrease in the expression level of GSK-3β. Concurrently, there was a notable increase in the level of phosphorylated GSK-3β (Ser9). Additionally, the expression of key proteins within the WNT/β-catenin pathway, specifically *β*-catenin and the GSK-3 downstream target c-MYC, was also significantly downregulated. These findings are depicted in Figure 4.

### 2.5 Effect of venetoclax combined with 9-ING-41 on the expression of apoptosis-related proteins in the DHL cell lines

Building upon the results of the previous Annexin V-FITC/PI double-staining flow cytometry experiment, which initially indicated that the combination of venetoclax and 9-ING-41 markedly augmented the proapoptotic impact of the two individual drugs on DHL cell lines, we sought to further validate this conclusion at the protein level. To this end, we treated the Karpas-422 cell line with 0.1% DMSO, 5 μM venetoclax, 0.5 μM 9-ING-41, or a combination of 5 μM venetoclax and 0.5 μM 9-ING-41. Similarly, the SuDHL2 cell line was treated with 0.1% DMSO, 10 μM venetoclax, 4 μM 9-ING-41, or a combination of 10 μM venetoclax and 4 μM 9-ING-41. Following a 48-hour incubation period, total protein was extracted from both cell lines, and the expression levels of the target proteins were analyzed using Western blot.

**Figure 5.**
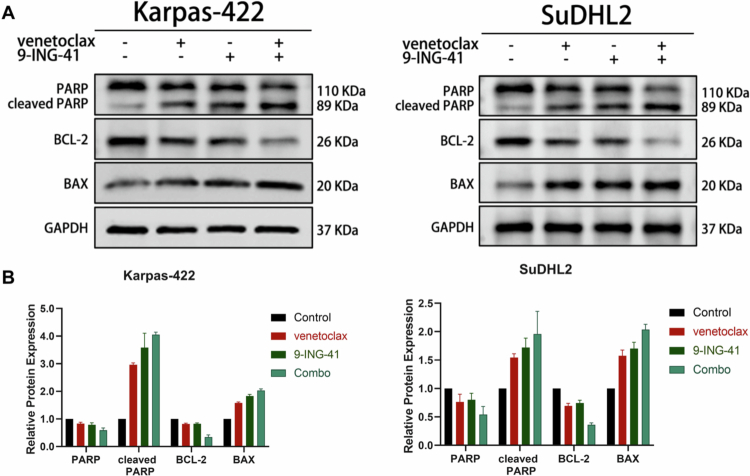
Effect of venetoclax combined with 9-ING-41 on the expression of apoptosis-related proteins in the DHL cell line. (A)(B) Western blot analysis of the effects of venetoclax, 9-ING-41 alone, and their combination on the expression levels of apoptosis-related proteins in the Karpas-422 and SuDHL2 cell lines.

The results demonstrated that for both the Karpas-422 and SuDHL2 cell lines, compared to the control group, the 9-ING-41 monotherapy group, the venetoclax monotherapy group, and the dual-drug combination group presented significant upregulation of the expression of the proapoptotic proteins cleaved PARP and Bax. Conversely, there was notable downregulation of the expression of the antiapoptotic proteins BCL2 and PARP. These findings further affirm the synergistic effect of venetoclax and 9-ING-41 in promoting the apoptosis of DHL cells, as depicted in Figure 5.

## 3 Discussion

DHL is characterized by its highly aggressive nature, susceptibility to drug resistance, and propensity for systemic and central nervous system relapses. Despite comprising only approximately 5% of diffuse large B-cell lymphomas (DLBCLs), DHL often responds poorly to conventional R-CHOP chemotherapy regimens, leading to a very unfavorable prognosis. The median overall survival (OS) in such cases can range from as short as 2.52 months to as long as 21.9 months.^13^^,^^14^ High-intensity chemotherapy regimens, such as DA-R-EPOCH, have improved PFS, with no difference in OS among DHL patients,^15^ but the clinical prognosis for this condition remains unsatisfactory. Hence, the research and development of precise targeted therapies tailored to specific gene variants in DHL are of paramount importance.

Among the targeted molecular inhibitors for DHL therapy, those targeting BCL2, c-Myc, BCL6, and immunosuppressants have garnered significant attention. Among these classifications, BCL2 inhibitors have received considerable attention and have been the first inhibitor class to enter clinical trials. Venetoclax has achieved a breakthrough in reducing platelet decline; however, prolonged use may still result in resistance among DHL cells.^16^

In recent years, notable advancements in the therapeutic targeting of c-Myc have been reported. Although no MYC inhibitors have been approved by the Food and Drug Administration (FDA) to date, these studies have provided invaluable insights into the development of novel therapeutic strategies.^17^^,^^18^ c-Myc is deemed an "undruggable" target owing to its status as a transcription factor with a relatively disordered domain and a lack of specific inhibition sites. Furthermore, its primary nuclear localization precludes the implementation of monoclonal antibody therapy. However, targeting c-Myc through alternative means, such as inhibiting its transcription, translation, stability, and transcriptional activity, has shown promising potential in antitumor therapy.^19^ Several clinical trials targeting c-Myc have been completed or are currently ongoing.^20^

The mechanisms through which GSK-3 inhibitors exert their effects in lymphoma cells include the induction of apoptosis via the downregulation of c-Myc signaling and the inhibition of cell proliferation by disrupting spindle function. Sahin et al. underscored the appeal of selective GSK-3β inhibitors as therapeutic targets, owing to their pivotal roles in regulating apoptosis, the cell cycle, DNA repair, tumor growth, invasion, and metastasis, as well as their capacity to modulate anticancer immune responses.^21^ Furthermore, Harrington et al. discovered that low-dose GSK-3 inhibitors (such as CHIR: LiCl), when combined with doxorubicin (Dox), could partially reverse Dox resistance in Ramos cell lines, potentially through the modulation of the WNT/β-catenin pathway.^22^ These studies underscore the potential of GSK-3 inhibitors in lymphoma treatment, particularly in the context of novel therapeutic strategies targeting specific molecular entities such as MYC and BCL2. The precise targeted therapy of DHL, particularly the combined use of BCL2 and Myc inhibitors, offers new hope for enhancing therapeutic outcomes in this refractory condition. With a deeper understanding of the molecular mechanisms underlying DHL, coupled with the clinical deployment of novel targeted medications, more effective treatments for DHL patients are anticipated.

In our study, we conducted an in-depth evaluation of the combined therapeutic potential of venetoclax (a BCL2 inhibitor) and 9-ING-41 (a GSK-3 inhibitor) in targeting DHL cell models. The experimental data demonstrated a significant synergistic effect between these two drugs in inhibiting DHL cell proliferation and promoting apoptosis when used in combination. However, Karmali et al. reported that the combination of 9-ING-41 and venetoclax did not confer significant benefit to Karpas-422 cells.^23^ In the study by Karmali et al., all lymphoma cell lines were tested at fixed concentrations (9-ING-41: 0–0.5 μM; venetoclax: 0–5000 nM). Our preliminary experiments revealed that the IC50 values for both drugs in Karpas-422 cells exceeded these ranges, suggesting that the concentrations that Karmali et al. employed may have been insufficient to reveal potential synergy. In preliminary experiments, we evaluated several different fixed ratios and identified the ratio that produced the strongest synergistic effect. Furthermore, we applied the Chou–Talalay mathematical model to quantitatively determine whether the combination exerted synergistic effects, thereby providing a more rigorous assessment of drug combinations.

Our findings revealed that the combination of venetoclax and 9-ING-41 significantly potentiated the cell cycle arrest effect, primarily by blocking the cell cycle at the G0/G1 phase. This result aligns with recent research that further elucidates the intricate roles played by c-Myc and GSK-3β in regulating cell cycle progression and maintaining chromosome stability. Specifically, Littler et al. noted that c-Myc overexpression can lead to chromosome separation defects and chromosomal instability by disrupting the microtubule core and spindle assembly, thereby promoting micronucleus formation—a marker of chromosomal instability—under the influence of antimitotic drugs.^24^ Additionally, the observations of Nilkhet S et al. emphasized that inhibiting AKT/GSK-3β phosphorylation may impact cell cycle regulation by stabilizing cytoplasmic and intracellular *β*-catenin, thereby playing a potential therapeutic role in anticancer therapy.^25^ These insights not only support our findings but also underscore the importance of therapeutic strategies aimed at promoting cell cycle arrest and synergistic cytotoxicity through the targeting of specific molecular mechanisms.

Western blot analysis in our study corroborated the effects of the venetoclax and 9-ING-41 combination on cells at the protein level, indicating that this drug duo may exert therapeutic benefits by modulating GSK-3β and its downstream WNT/β-catenin signaling pathway. This discovery resonates with previous findings and provides a scientific rationale for targeting GSK-3β and its associated pathways in the treatment of DHL. Studies have shown that GSK-3β inhibition impedes the phosphorylation and degradation of *β*-catenin, leading to increased nuclear accumulation of *β*-catenin, which in turn activates the WNT/β-catenin pathway and promotes the expression of genes related to cell survival.^26^ The activation of the WNT/β-catenin signaling pathway has been demonstrated to play a crucial role in the development and progression of multiple cancers, including DHL.^27^

Despite progress in revealing the synergistic effect of venetoclax and 9-ING-41 on DHL cell lines, this study has several limitations. Notably, these findings at the cellular level require in vivo validation in animal models. Further research is needed to clarify the specific molecular mechanisms, especially their impact on other DHL signaling pathways.

Future studies should investigate the effects of this drug combination across diverse DHL models, with a particular focus on its potential to overcome resistance and improve patient survival. Comprehensive pharmacokinetic and pharmacodynamic evaluations of 9-ING-41 and venetoclax in clinical settings are also warranted to establish clinically achievable doses and exposure times. Moreover, in vivo validation and direct comparisons with current chemotherapy and targeted regimens will be essential to fully assess the therapeutic potential of this combination in the context of standard DHL treatment.

## 4 Materials and methods

### 4.1 Cell culture and reagents

DHL cell lines Karpas-422 and SUDHL2 (human, Myc and BCL2 rearrangements) were obtained from ATCC. The cells were maintained in RPMI-1640 medium (Procell, PM150110) supplemented with 10% fetal bovine serum (Gibco, #10270-106) and 1% penicillin/streptomycin (Gibco, #15140122) at 37 °C in a 5% CO₂ incubator.

Reagents 9-ING-41 (MCE, #HY-113914-10 mg) and venetoclax (MCE, #HY-15531-10 mM/1mL) were dissolved in dimethyl sulfoxide to generate 50 mM stock solutions, which were filtered (0.22 μm), aliquoted, and stored at -80 °C. The working concentrations were diluted in complete medium.

### 4.2 Cell viability and proliferation assays

For the CCK-8 assay, cells (Karpas-422: 1 × 10⁶/mL; SUDHL2: 5 × 10⁵/mL) were seeded in 96-well plates and treated with 0.25–2 μM (Karpas-422) or 2–10 μM (SUDHL2) 9-ING-41, 5–30 μM venetoclax, or combinations (Table 1). After 24 or 48 hours, 10 μL of CCK-8 reagent (APExBIO, #K1018-20) was added. The absorbance at 450 nm was measured using a microplate reader (Shenzhen Huisong Technology, China).

**Table 1.** Establishment of drug combination ratios and concentration gradients based on the proliferation inhibition rate and IC_50_ of single drugs on DHL cell lines (Karpas-422, SuDHL2).

	Karpas-422		SuDHL2	
	24 H	48 H	24 H	48 H
9-ING-41 (μM)/venetoclax (μM)	0.05/2.5 0.1/5 0.2/10 0.3/15 0.4/20 0.5/25 0.6/30	0.1/1 0.25/2.5 0.5/5 0.75/7.5 1/10 1.5/15 2/20	1/3 2/6 3/9 4/12 5/15 6/18 7/21	2/5 4/10 6/15 8/20 10/25 12/30 14/35
9-ING-41:venetoclax	1:50	1:10	1:3	1:2.5

Analysis: Cell viability (%) = [(ODtreatment − ODblank)/(ODcontrol − ODblank)] × 100. Cell inhibition rate (%) = 1 − viability (%). IC50-based analyses and the CI method were used to assess drug efficacy and synergy. IC50 values and combination indices (CI) were calculated using GraphPad Prism 9.0 and CompuSyn.

### 4.3 Analysis of the cell cycle and apoptosis

Apoptosis: After 48 hours of treatment, the cells were stained with Annexin V-FITC/PI (KeyGEN BioTECH, #KGA108) and analyzed using a BD FACSCalibur flow cytometer (BD Biosciences, USA). The data were processed with FlowJo V10.

Cell cycle: Ethanol-fixed cells were stained with PI/RNase solution (Multisciences, #CCS012). Sub-G₁ populations were quantified via flow cytometry.

### 4.4 Western blotting

Protein extraction: Cells were lysed in RIPA buffer (Fdbio Science, #FD009) containing protease/phosphatase inhibitors (Fdbio Science, #FD1002). Lysates were centrifuged at 12,000 × g for 15 minutes, and protein concentrations were determined via a BCA assay (Fdbio Science, #FD2001).

Electrophoresis and immunoblotting: Proteins (30 μg per lane) were separated on 10% SDS‒PAGE gels (EpiZyme, #PG112), transferred to PVDF membranes (Millipore, #IPVH00010), and probed with primary antibodies (1:1,000; #Cell Signaling Technology, USA) and HRP-conjugated secondary antibodies (1:5,000; CST, USA). The blots were visualized using ECL substrate (Glpbio, #GK10008). The following primary antibodies were used: GSK-3β (1: 2,000; CST, #12456T), phospho-GSK-3β (Ser9) (D85E12) (1:1,000; CST, #5558T), *β*-catenin (D10A8) (1:2,000; CST, #8480T), c-Myc/N-Myc (D3N8F) (1:2,000; CST, #13987T), PARP (46D11) (1:2,000; CST, #9532T), Bax (D2E11) (1:2,000; CST, #5023T), Bcl2 (1:2,000; Proteintech, #26593-1-AP-50UL) and GAPDH (1:5,000;CST, #5174T).

### 4.5 Statistical analysis

Data analysis was performed using SPSS 25.0 and GraphPad Prism 9.0. For homogeneous variances, two-sample t-tests were used to compare two groups, and one-way ANOVA was employed to assess multiple groups. The post hoc LSD method was applied for significant ANOVA results. For heterogeneous variances, an approximate F-test preceded Dunnett’s T3 or C methods for pairwise comparisons. Skewed data were reported as the median ± interquartile range and were analyzed using non-parametric tests. Statistical significance was set at *P* < 0.05, denoted as *P < 0.05, **P < 0.01, ***P < 0.001, ****P < 0.0001. All the experiments were carried out in triplicate.

## Data Availability

All the data generated and analyzed throughout this study are included in the article. All the data and material in this paper are available when requested.
